# Is a woolen cap effective in maintaining normothermia in low-birth-weight infants during kangaroo mother care? Study protocol for a randomized controlled trial

**DOI:** 10.1186/s13063-016-1387-0

**Published:** 2016-05-26

**Authors:** Daniele Trevisanuto, Giovanni Putoto, Damiano Pizzol, Tiziana Serena, Fabio Manenti, Silvia Varano, Eleonora Urso, William Massavon, Ademe Tsegaye, Oliver Wingi, Emanuel Onapa, Giulia Segafredo, Francesco Cavallin

**Affiliations:** Department of Women and Children Health, University of Padua, Azienda Ospedaliera di Padova, Via Giustiniani, 3, 35128 Padova, Italy; Doctors with Africa CUAMM, Padova, Italy; Doctors with Africa CUAMM, Central Hospital of Beira, Beira, Mozambique; Department of Pediatrics, University of Bari, Bari, Italy; Doctors with Africa CUAMM, Oyam District, Uganda; Doctors with Africa CUAMM, Wolisso, Ethiopia; Department of Pediatrics and Neonatology, Central Hospital of Beira, Beira, Mozambique; Hospital of Aber, Aber, Uganda; Padova, Italy

**Keywords:** Cap, Kangaroo mother care, Temperature, Newborn infant

## Abstract

**Background:**

Neonatal hypothermia is an important challenge associated with morbidity and mortality. Preventing neonatal hypothermia is important in high-resource countries, but is of fundamental importance in low-resource settings where supportive care is limited.

Kangaroo mother care (KMC) is a low-cost intervention that, whenever possible, is strongly recommended for temperature maintenance. During KMC, the World Health Organization (WHO) guidelines recommend the use of a cap/hat, but its effect on temperature control during KMC remains to be established.

In the hospitals participating in the projects of the non-governmental organization CUAMM, KMC represents a standard of care, but the heads of the babies often remain uncovered due to local habits or to the unavailability of a cap. The aim of the present study will be to assess the effectiveness and safety of using a woolen cap in maintaining normothermia in low-birth-weight infants (LBWI) during KMC.

**Methods/design:**

This is a multicenter (three hospitals), multicountry (three countries), prospective, unblinded, randomized controlled trial of KMC treatment with and without a woolen cap in LBWI. After obtaining parental consent, all infants with a birth weight below 2500 g and who are candidates for KMC, based on the clinical decision of the attending physician, will be assigned to the KMC with a woolen cap group or to the KMC without a woolen cap group in a 1:1 ratio according to a computer-generated, randomized sequence. The duration of the study will be until the patient’s discharge, with a maximum treatment duration of 7 days.

The primary outcome measure will be whether the infants’ temperatures remain within the normal range (36.5–37.5 °C) in the course of KMC during the intervention. In all participants, axillary temperature will be measured with a digital thermometer four times per day. In addition, maternal and room temperature will be recorded.

Secondary outcome measures will be: episodes of apnea; sepsis; mortality before hospital discharge; in-hospital growth; and age at discharge.

**Discussion:**

The findings of this study will be important for other units/settings in high- as well low-resource countries where KMC is routinely performed. Based on the results of the present study, we could speculate whether the use of a woolen cap may help to maintain the neonate within the normal thermal range. Furthermore, potential complications such as hyperthermia will be strictly monitored and collected.

**Trial registration:**

ClinicalTrials.gov Identifier: NCT02645526 (registered on 31 December 2015).

## Background

The days and weeks following childbirth – the postnatal period – is a critical phase in the lives of mothers and newborn babies. Most maternal and infant deaths occur during this time [1]. In this period, neonatal hypothermia is an important challenge associated with morbidity and mortality [2]. Hypothermia increases the newborn’s metabolic requirements and is associated with hypoglycemia, hypoxia, and ultimately severe infections and newborn mortality [3]. Preventing neonatal hypothermia is important in high-resource countries, but is of fundamental importance in low-resource settings where supportive care is limited.

Kangaroo mother care (KMC) is a low-cost intervention that has the potential to prevent many complications associated with preterm birth and may also provide benefits to full-term newborns [4].

KMC is care of a small baby who is continuously carried in skin-to-skin contact by the mother and exclusively breastfed (ideally). It is considered the best way to keep a small baby warm and it also helps to establish breastfeeding. KMC can be started in the hospital as soon as the baby’s condition permits (i.e., the baby does not require special treatment, such as oxygen or intravenously administered fluids) [5].

Whenever possible, KMC is strongly recommended for temperature maintenance [4]. During KMC, the World Health Organization (WHO) guidelines recommend the following approach: “clothe the baby with a pre-warmed shirt open at the front, a napkin, a hat, and socks” [5].

Previous studies show that neonatal heat loss following delivery may be reduced or prevented by the application of simple woolen hats [6–8], but the effect of a cap on thermal control of neonates during the first postnatal days remains to be defined. On the other hand, hyperthermia should also be avoided [9, 10].

Doctors with Africa CUAMM is a non-governmental organization supporting health programs in different African countries. In the hospitals participating in the CUAMM projects, KMC represents a standard of care, but the heads of the babies often remain uncovered due to local habits or, more simply, to the unavailability of a cap. However, although WHO guidelines recommend the use of a cap/hat during KMC, the effect on neonatal temperature during the days and weeks following childbirth has not been previously studied. It is unknown whether covering the head of the neonate with a woolen cap during KMC may help temperature maintenance. The results of the present study will allow us to understand whether the use of a cap during KMC is effective and safe.

Previous studies suggest that neonatal heat loss following delivery may be reduced or prevented by the application of a woolen hat [6–8]. These studies assessed patients immediately after birth and for a short period. On the other hand, hyperthermia can be dangerous to the newborn infant and should also be avoided [9, 10].

To our knowledge, there are no previously published studies that have evaluated the effect of using a woolen cap in the course of KMC.

The aim of the present study will be to assess the effectiveness and safety of using a woolen cap in maintaining normothermia in low-birth-weight infants (LBWI) during KMC.

## Methods/design

### Study design

This is a multicenter, prospective, unblinded, randomized clinical trial of KMC treatment with and without a woolen cap in LBWI.

### Setting

The study will be conducted at three hospitals that have three different levels of healthcare in three African countries where Doctors with Africa CUAMM has ongoing projects on maternal-neonatal health. They are:

1. Central Hospital of Beira in Mozambique (5555 deliveries – CUAMM data 2013), which is a governmental hospital (III level) (Mozambique: neonatal deaths, 34 % of all under-5 deaths; neonatal mortality rate: 30 per 1000 live births; source: *Countdown to 2015, The 2014 Report*) (http://www.mediciconlafrica.org content/uploads/sites/2/2015/09/Health_and_Devel_71_giu15-BASSA.pdf)
2. St. Luke Wolisso Hospital in Ethiopia (3323 deliveries – CUAMM data 2013), which is a no-profit zonal hospital (II level) (Ethiopia: neonatal deaths, 43 % of all under-5 deaths; neonatal mortality rate: 29 per 1000 live births; source: *Countdown to 2015, The 2014 Report*) (http://www.mediciconlafrica.org content/uploads/sites/2/2015/09/Health_and_Devel_71_giu15-BASSA.pdf)
3. Aber Hospital in Uganda (1872 deliveries – CUAMM data 2013), which is a no-profit rural hospital (I level) (Uganda: neonatal deaths, 33 % of all under-5 deaths; neonatal mortality rate 23 per 1000 live births; source: *Countdown to 2015, The 2014 Report*) (http://www.mediciconlafrica.org content/uploads/sites/2/2015/09/Health_and_Devel_71_giu15-BASSA.pdf)

### Inclusion criteria

Infants satisfying the following inclusion criteria will be eligible to participate in the study:

1. Birth weight below 2500 g (and)
2. Candidate to KMC treatment (and)
3. Parental consent: a written informed consent will be obtained by a member of the neonatal team involved in the study from a parent or guardian before KMC treatment

### Exclusion criteria

1. Major congenital malformations
2. Twins
3. Parental refusal to participate in the study

### Procedure

After obtaining parental consent, all infants with a birth weight below 2500 g and who are candidates for KMC, based on the clinical decision of the attending physician, will be assigned to the KMC with a woolen cap group (KMC + cap) or to the KMC without a woolen cap group (KMC group). All the caps used for the study are handmade by a group of volunteers in Padova. The material (wool) was provided by CUAMM and sizes were standardized.

The procedure of initiating, maintaining and stopping the KMC will be based on WHO guidelines [5]. Mothers will be trained in monitoring the baby’s condition and in recognizing dangerous clinical signs during KMC [5]. The duration of the study will be until the patient’s discharge, with a maximum treatment duration of 7 days. In case of documented neonatal hypothermia (temperature below 36.5 °C) or hyperthermia (temperature above 37.5 °C) during the course of the study, the pre-warmed blanket will be added or removed. In case of maternal hyperthermia, we will ask the woman to wear lighter clothes. In these cases, neonatal and maternal temperatures will be measured every hour until the normal range is reached.

### Primary outcome measure

The primary outcome measure will be whether the infants’ temperatures remain within the normal range (36.5–37.5 °C) in the course of KMC during the intervention. In all participants, axillary temperature will be measured with a digital thermometer (C202; Terumo, Tokyo, Japan) every 6 hours during KMC treatment. Maternal and room temperature will be registered at the same time. Room temperature will be measured at the same times (every 6 hours) by using the same wall thermometers (Oregon Scientific RMR262) in all three study sites.

### Secondary outcome measures

1. Episodes of apnea, defined as the need for sustained stimulation to initiate breathing
2. Sepsis, defined as the presence of at least two of the following clinical signs: lethargy, persistent apnea, poor feeding; fever
3. Mortality before hospital discharge
4. In-hospital growth, defined as weight at discharge

### Generalizability

The findings of this study will be important for other units/settings in high- as well low-resource countries where KMC is routinely performed. Based on the results of the present study, we could speculate whether the use of a woolen cap may help to maintain the neonate within the normal thermal range. Furthermore, potential complications such as hyperthermia will be strictly monitored and collected.

### Sample size

The lack of information about this topic (temperature during KMC in the specific scenario of head covering) prevented us from obtaining a mathematical estimation of the sample size. Thus, the sample size was arbitrarily defined in order to take into account the enrollment rate of each hospital. According to the number of admissions in each participating hospital, we decided to enroll a total of 300 subjects (150 in the cap group and 150 in the uncovered head group) as follows: 150 at Central Beira Hospital, 90 at St. Luke Wolisso Hospital and 60 at Aber Hospital.

### Recruitment

Written and oral information will be offered to parents by the attending nurse or physician prior to starting KMC treatment. Informed written consent will be signed by a parent. A senior investigator will be available at all times to discuss concerns raised by parents or clinicians during the course of the trial.

### Randomization

Eligible infants will be assigned to the KMC + cap or the KMC group in a 1:1 ratio according to a computer-generated, randomized sequence for each participating hospital. The randomized allocation will be concealed in double-enclosed, opaque, sealed, and sequentially numbered envelopes prepared at the University Hospital of Padua.

**Fig. 1:**
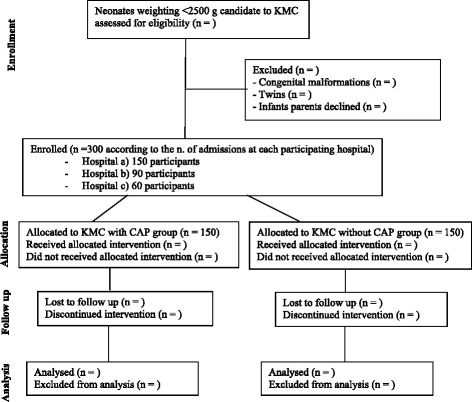
Flow diagram of patient randomization

In the KMC room, the next sequential randomization envelope will be opened only when the attending operator considers the infant to be eligible. The assigned procedure (KMC + cap or KMC group) will be then performed. Cross-over will not be allowed. Analysis will be done as per intention-to-treat. A flow diagram of patient randomization is reported in Fig. 1.

### Blinding

Due to the characteristics of the intervention, neither caregivers nor outcome assessors will be masked to treatment allocation. However, the statistician who will perform data analysis will be blind to treatment allocation. To minimize bias, strict criteria and definitions will be maintained during the trial.

### Guidelines for management

Before starting the study, all those involved in the study will participate in a meeting where all the details of the study will be presented. Midwives and nurses who are responsible of the maternal and neonatal management during KMC treatment will be educated on the temperature measurements in terms of correct modality (axillary), time of the study, and data collection. Neonatal, maternal and room temperature will be collected four times/day (6.00 am, 12.00 am, 6.00 pm, and 12.00 pm).

### Data collection

Data will be recorded in a data sheet designed for this study. All data will be collected by an observer who is not involved in the care of the neonates. The following clinical information will be registered: eligibility, antenatal history, randomization, all data above listed in the ‘Primary outcome measure’ and ‘Secondary outcome measures’ sections. Further information will be collected on expected serious adverse events (SAEs). Personal information about potential and enrolled participants will be collected, shared, and maintained in order to protect confidentiality before, during, and after the trial by the local principal investigator (PI) in a personal password-protected PC. The PI and the members of the Steering Committee located in Padua will have access to the final trial dataset.

### Statistical analysis

Analysis will be performed as per intention-to-treat. Continuous data will be expressed as mean and standard deviation (SD) or median and interquartile range (IQR). The effect of the treatment on primary outcome will be assessed using a Poisson model adjusting for hospital and a set of clinically relevant confounders (maternal temperature, environmental temperature, birth weight, postnatal age). The logarithm of the number of measurements during hospital stay will be used as an offset, in order to take into account the different lengths of hospital stay of each participant. The effect of the treatment on the number of episodes of apnea will be assessed using a Poisson model adjusting for hospital and a set of clinically relevant confounders (neonatal and maternal temperatures, environmental temperature, birth weight, postnatal age). The logarithm of the number of measurements during hospital stay will be used as an offset. The effect of the treatment on binary secondary outcome (sepsis and mortality before hospital discharge) will be assessed using a logistic regression model adjusting for hospital and birth weight. The effect of the treatment on continuous secondary outcome (in-hospital growth) will be assessed using a regression model adjusting for hospital. Statistical analysis will be performed using R 3.2.2 software (R Foundation for Statistical Computing, Vienna, Austria) [11]. A *p* value less than 0.05 will be considered statistically significant.

### Duration of study

In this study, 300 infants will be recruited. The trial will terminate when the last recruited infant is discharged from hospital, or dies.

Based on a recent study conducted by our group at the Central Hospital of Beira, Mozambique, we consider that we will perform the study in about 14 months (Table 1).

**Table 1:** Duration of the study

Time	2 months	3 months	6 months	1 month	2 months
Task	Preparation of the protocol and Case Report Form (CRF), and material (i.e.. thermometer, woolen cap); (completed)	Ethics and Ministerial Committee approval; (completed)	Data collection	Analysis of the data	Preparation of the manuscript
Done	x	x	x (started in January 2016)		

The study will be conducted – simultaneously – at the three participating hospitals.

### Ethical considerations

Written parental consent is necessary before enrollment of the patients in the study. We consider that there will be no risks for either study group (patients treated with or without a woolen cap). Hypothermic as well as hyperthermic episodes will be strictly monitored in both groups.

### Ethics Committee approval

The study was approved by the Ethics Committees for Human Investigation of the three participating hospitals. At the Central Hospital of Beira the study was approved by the Comitè Nacional di Bioetica para a Saude, Ministerio da Saude (REF: 348/CNBS/15: IRB00002657; Maputo, Mozambique; 8 December 2015); at St. Luke Wolisso Hospital the study was approved by the Lacor Hospital Institutional Research and Ethics Committee (REF: LHIREC N.: 016/03/15; Gulu, Uganda; 30 May 2015); at Aber Hospital the study was approved by the Oromia Regional Health Research Ethical Review Committee Bureau (REF: BEFOABTFH 3657/1-8; Addis Ababa, Ethiopia; 24 June 2015).

### Compliance to protocol

Compliance will be defined as full adherence to protocol. Compliance with the protocol will be ensured by some members of the project (DM, TS, SM, WM) who are responsible for local data collection. They will monitor adherence to the study protocol weekly and will input the patients’ data in an Excel data sheet. Double data entry will be performed by two independent members at each center to promote data quality.

### Missing data

Investigators and study staff will be trained on the importance of completion of the study period of enrolled patients. Parents will also be informed about this crucial aspect to reduce dropout, and a local investigator will be available at each site any time that the parents may need further information or clarification during the study period.

### Data Safety and Monitoring Board

Safety measures will include incidence, severity and causality of reported SAEs, represented by changes in occurrence of the expected common neonatal complications and the development of unexpected SAEs. SAE will be defined as unexpected death, apnea not responding to vigorous stimulation, severe hyperthermia (temperature >39 °C) and hypothermia (temperature <35 °C). All SAEs will be followed until complete resolution or until the clinician responsible for the care of the recruited patient considers the event to be chronic or the infant to be stable.

A monitoring board, including an independent assessor (not involved in the study) from the University of Padova and assessors from each participating hospital, will review all the deaths and adverse events. If there is a reasonable suspected causal relationship with the intervention, SAEs will be reported to the Ethics Committee to guarantee the safety of the participants.

An interim analysis will be performed on the primary endpoint and on SAEs from the first 100 enrolled infants. The interim analysis will be performed by the statistician, who is blinded for the treatment allocation and who will report to the PI. The PI will discuss the results of the interim analysis with the monitoring board and the trial will be ended in case of harm. Criteria for stopping for harm include: a statistically significant difference in the primary outcome between the treatment groups; and a reasonable suspected causal relationship between the intervention and SAEs.

### Confidentiality

Only the local PI will have access to the Excel database with an assigned personal account and password. Subjects will be identified by sex, birth date, and assigned trial number, during and after the trial, in accordance with personal data protection law.

### Access to data

The PIs of each site will have complete access to the final trial dataset, and no contractual agreement exists to limit such access for the investigators.

### Dissemination policy

The results of the trial are expected to be published in a scientific journal and presented at medical seminars and conferences. The final reporting will follow the Consolidated Standards of Reporting Trials (CONSORT) Statement guidelines (http://www.consort-statement.org).

## Discussion

There are unique features of this trial compared to prior studies on KMC. World Health Organization guidelines recommend the use of a cap during KMC treatment [5], but evidence for this practice is lacking. In this trial, we will assess the efficacy and safety of using a woolen cap during KMC treatment.

### Trial status

The trial was approved by the Ethics Committees of the participating hospitals. Enrollment of patients started in January 2016.

## Abbreviations

CRF: Case Report Form
KMC: kangaroo mother care
LBWI: low-birth-weight infant
SAE: serious adverse event
WHO: World Health Organization
